# Face Covered and Six Feet Apart: Behavioral Awareness Predicts Greater Adherence to Public Health Guidelines during the COVID-19 Pandemic

**DOI:** 10.3390/ijerph18168247

**Published:** 2021-08-04

**Authors:** Alyssa Schneider, Emily B. Kroska

**Affiliations:** Department of Psychology and Brain Sciences, University of Iowa, Iowa City, IA 52242, USA; alyssa-schneider@uiowa.edu

**Keywords:** COVID-19, Acceptance and Commitment Therapy, psychological flexibility, masks, behavioral awareness

## Abstract

The COVID-19 pandemic has deleteriously impacted physical and mental health. Guidelines to limit the spread of COVID-19 include wearing a face covering in public, limiting close contacts, and physical distancing. In combatting this and future pandemics, it is essential to understand predictors of adherence, such as psychological flexibility. We hypothesized higher psychological flexibility would relate to greater adherence to public health guidelines. Participants (*n* = 265) were English-reading/speaking adults in the United States and were recruited through Amazon’s Mechanical Turk. Included in the present analyses are data from June (*n* = 360) and July 2020 (*n* = 265). Measures included the Comprehensive Assessment of ACT Processes (CompACT), which measured psychological flexibility. Outcome measures included mask-wearing and number of close contacts, which were operationalized categorically (100% mask-wearing in public, ≤10 close contacts in past week). Two logistic regression models examined psychological flexibility and distress as predictors of adherence to mask-wearing and limiting close contacts, while controlling for demographic correlates. Results indicated that greater behavioral awareness predicted greater odds of mask-wearing and limiting close contacts. Psychological flexibility, and behavioral awareness specifically, should be investigated in future research as targets for intervention amidst global disasters.

## 1. Introduction

The COVID-19 pandemic has affected the world drastically, bringing with it sickness and the deaths of millions of people. In December of 2019, the first cases of COVID-19 were seen in the Wuhan province of China, and by January of 2020, COVID-19 had spread throughout the world, including the United States [1]. Shortly thereafter, COVID-19 was declared a pandemic and a national emergency in the United States [1]. The pandemic was associated with a multitude of negative outcomes in terms of physical and mental health [2,3]. With respect to mental health, lower psychological well-being, and higher anxiety and depression, were reported when compared to pre-pandemic levels [4]. With a costly death toll, public health guidance was central to slow the spread of the coronavirus.

Public health guidelines played a key role in combatting the community spread of COVID-19, including handwashing/sanitizing, wearing a face (mouth and nose) covering when in public, limiting close contacts with others (keeping a distance of at least six feet from others), and avoiding public gatherings (i.e., not going to non-essential events/areas that could contribute to the spread of COVID-19) [5]. However, resistance to these public health measures was (and remains) notably strong in the United States [6]. Understanding predictors of increased adherence with these public health measures is therefore essential for the COVID-19 pandemic, but also for future pandemics.

Given the widespread psychosocial and health impacts of COVID-19, it is important to identify modifiable transdiagnostic processes; that is, processes that are applicable across diagnoses that can be targeted in future intervention and prevention research. In the United States, China, and Germany, researchers found that high levels of psychological distress were associated with COVID-19, and have emphasized the importance of understanding the psychological processes underlying distress [7,8]. A transdiagnostic process that centers on a person’s response to distress—psychological flexibility—has demonstrated associations with distress, as well as other adverse psychological outcomes [9]. Psychological flexibility consists of three components: behavioral awareness, openness to experiences, and engagement [9]. Openness to experiences describes a willingness to have uncomfortable internal experiences when related to personal values; behavioral awareness describes intentionally paying attention to internal experiences, context, and behavior; and engagement describes making choices that align with personally chosen values [9]. In a study that focused on psychological flexibility in the context of COVID-19, researchers found that participants that scored lower on components of psychological flexibility, including openness to experiences and behavioral awareness, were also found to experience higher general distress [10]. During the nationwide lockdown in Italy, researchers examined the impact of psychological flexibility on health anxiety and found that psychological flexibility mitigated the detrimental effects of the lockdown on mental health [11]. Although psychological flexibility has been investigated in the context of mental health during the pandemic, it has not been examined as a predictor of public health behaviors that impact global communities. This is particularly important given the implications for the most vulnerable in these communities. Understanding psychological flexibility, along with psychological distress, may provide important insights related to adherence with public health guidelines aimed to slow the spread of COVID-19.

### Objectives of the Present Study

The current study aimed to investigate predictors of adherence with public health guidelines (mask-wearing, limiting close contacts), including psychological distress and psychological flexibility. We hypothesized that higher levels of psychological flexibility and lower levels of distress would predict greater adherence with public health guidelines. Because psychological flexibility is related to other health behaviors, such as adherence to medical regiments or weight loss, we anticipated that psychological flexibility would be associated with greater adherence to public health guidance during the COVID-19 pandemic [12].

## 2. Materials and Methods

### 2.1. Participants

Participants (*n* = 265) were English-reading/speaking adults in the United States. The average age of the sample was 38.49 years (SD = 11.42). The majority of the sample identified as of White race (78.8%) and reported being employed full-time (75.4%). The number of participants reported represents the number of participants who completed the third and final assessment in the longitudinal study where data were collected in May, June, and July of 2020. Attrition analyses below examined differences in demographic factors between those who completed all study timepoints and those who did not. See Table 1 for further demographic information.

### 2.2. Procedures

Participants were recruited via Mechanical Turk’s CloudResearch platform, an online service in which tasks, called HITs (Human Intelligence Tasks), are made available to registered users. Given established concerns with inattentive and non-human responders, authors took several measures to ensure human participants and to screen for attentive responding. Completion by participants in suspicious geolocations was prevented by CloudResearch. Participants enrolled through Amazon’s Mechanical Turk, where they were required to have completed at least 100 HITs and to have at least a 95% approval rating in order to establish a record of reliable completion. Participants who met eligibility criteria selected the study from a list of available HITs and were provided with a link to the survey through CloudResearch, which was hosted through Qualtrics, a secure survey platform. The first page of the survey was a consent letter notifying participants that if they clicked forward, they were indicating their consent to participate. Written informed consent was waived, and approval was received by the University IRB. Participants completed a series of assessments on Qualtrics in May of 2020 for the baseline assessment (*n* = 485). This was followed by two surveys in June and July 2020. Voluntary participation was emphasized at each time point with a consent summary. The outcome variables in the present analyses were collected in July 2020 (*n* = 265), and predictors were collected in June 2020 (*n* = 360); 95 participants dropped out between time points. Within the survey, attention check items were included to screen for automatic or inattentive responding. Participants that answered attention checks incorrectly were not included in the analyses. A Google reCAPTCHA item was the first question following consent, and if incomplete, the survey would not proceed. Finally, an open-format arithmetic question required text input, another measure taken to prevent non-human participation. Participants were compensated for participation in each of the surveys.

### 2.3. Measures

#### 2.3.1. Distress

The Perceived Stress Scale-10 (PSS-10) was used to measure the perception of stress in the last month in June of 2020 [13]. Using a 5-point Likert scale (0 = Never; 4 = Very Often), participants rated the frequency of overwhelmed feelings. Items are summed for the total score, with higher scores indicating higher distress (range: 0–40). Face and content validity, as well as internal consistency, have been demonstrated in a prior work [13]. Internal consistency in the current sample was adequate (α = 0.96).

#### 2.3.2. Psychological Flexibility

The Comprehensive Assessment of Acceptance and Commitment Therapy processes (CompACT) is a 23-item measure of psychological flexibility and has three subscales: openness to experiences, behavioral awareness, and valued action [14]. Openness to experiences describes a willingness to have uncomfortable internal experiences, behavioral awareness describes intentionally paying attention to one’s own behavior, and valued action describes engaging in behavior that aligns with what a person deems important in their life [14]. The scale has demonstrated concurrent, convergent, and discriminant validity, as well as internal consistency [14]. Internal consistencies of the subscales in the current sample were adequate (openness to experiences α = 0.85, behavioral awareness α = 0.92, valued action α = 0.90). Psychological flexibility components were measured in June 2020.

#### 2.3.3. Adherence to COVID-19 Public Health Behaviors

The present study measured two public health behaviors that were emphasized strongly in the United States: (1) wearing a face covering in public, and (2) limiting close contact (within 6 feet) with others, and both adherence behaviors were measured in July 2020. Participants were asked to report the percentage of time that they wore a mask in public places, ranging from 0–100%. Given that mask-wearing was recommended in public places 100% of the time by the CDC and WHO, mask-wearing was operationalized as whether a person reported wearing a face mask 100% of the time in public places, and responses were coded categorically. Close contacts were assessed with a question reading “In the last week, how many people have you come in close contact with (within 6 feet)? This includes relatives, neighbors, delivery workers, shoppers, or strangers.” For the current analyses, data were recoded categorically to represent 10 or fewer contacts in the past 7 days, as compared to 11 or more contacts. The reason for this split between 10 or fewer contacts and 11 or more contacts was due to the CDC guidelines established in 2020 that advised limiting gatherings to 10 or fewer individuals [5].

##### Data Analyses

Attrition analyses were conducted to examine whether completion status related to demographic variables at baseline. Preliminary analyses were conducted to ascertain the relation of demographic variables and adherence with public health variables using independent samples *t*-tests and Chi-square tests of mean difference. The demographic variables assessed included gender identity, age, education, race, and ethnicity, which were measured at baseline. Other covariates included household income and relationship status, which were measured at the third and final assessment in July 2020.

Initial a priori power analyses for the larger longitudinal study were completed to conduct mediation and regression analyses. For regression analyses, G*Power 3.1.9.4 was utilized to determine what sample size was needed. G*Power is a freely available software program that is used to calculate statistical power across a number of effect sizes. Results indicated that for change in R^2^ with 6 predictors, a moderate effect size, and 0.80 power, a sample of 85 was needed.

Binary logistic regression analyses were utilized to examine predictors (psychological flexibility, distress, covariates) of adherence to public health recommendations, as measured in July 2020, one month following the measurement of psychological flexibility and distress (June 2020). The three psychological flexibility components (openness to experiences, behavioral awareness, and valued action) were included, rather than the total score, to determine if any of the components were more predictive of adherence while controlling for shared variance. Analyses included Nagelkerke’s Pseudo-R^2^ to assess the amount of variance accounted for in the outcome variable by the predictors.

## 3. Results

### 3.1. Preliminary Analyses

#### 3.1.1. Attrition Analyses

We examined whether those who completed all three time points of the study differed from those who dropped out of the study. Age differed by completer status (*t*(482) = −2.26, *p* = 0.03), such that completers (*M* = 38.49, *SD* = 11.42) were likely to be older than non-completers (*M* = 36.14, *SD* = 11.40). Years of education also differed by completer status (*t*(394.53) = −2.15, *p* = 0.03), such that completers had more years of education (*M* = 15.35, *SD* = 2.53) than non-completers (*M* = 14.76, *SD* = 3.33). Gender identity was not related to completion status (*X*^2^(1) = 0.12, *p* = 0.91). Ethnicity was related to completion status (*X*^2^(1) = 19.21, *p* ≤ 0.001), such that a smaller proportion of individuals identifying as Hispanic/Latinx (32.5%) completed the study than the proportion of non-Hispanic/Latinx self-identified individuals who completed (59.2%). Race, categorized as White/non-White due to lower numbers of other races, was not related to completion status (*X*^2^(1) = 1.95, *p* = 0.75). Income and relationship status were not measured at baseline, and thus could not be included in these comparisons.

#### 3.1.2. Mask Adherence

Gender identity was examined categorically due to limited participants reporting transgender or queer identities. Male and female gender identity were examined in relation to mask adherence, with females (63.5%) self-reporting greater mask adherence, *Χ*^2^(1) = 8.05, *p* = 0.005, than males (45.6%). Age and education were examined in relation to adherence with public health guidance with independent samples *t*-tests. Older-aged individuals self-reported greater mask adherence (*M* = 39.83, *SD* = 11.77) than those that were younger (*M* = 37, *SD* = 10.88), *t*(263) = −2.03, *p* = 0.04. No differences were observed with regard to years of education between those who reported mask-wearing and those who did not, *t*(260) = −0.68, *p* = 0.49.

Race was examined categorically by comparing White to non-White (African American, Black, American Indian, Alaska Native, Asian, Native Hawaiian, Pacific Islander, biracial, or multiracial) individuals, given lower frequency of reported races other than White. No differences were observed between White and non-White individuals in mask adherence, *Χ*^2^(1) = 0.2, *p* = 0.65. No differences were observed between non-Hispanic/Latinx and Hispanic/Latinx individuals in terms of mask adherence, *Χ*^2^(1) = 0.06, *p* = 0.81.

Household income was examined categorically by comparing those who reported having a combined household income below USD 70,000 to those who reported having a combined household income above USD 70,000, as the median in this sample was in the USD 50,000–70,000 category, and the national average household income reported in March of 2019 was USD 68,703 [15]. No differences were observed between those who reported having a combined household income below USD 70,000 and those who reported having a combined household income above USD 70,000 in mask adherence, *Χ*^2^(1) = 0.44, *p* = 0.51. No differences were observed between those in a romantic relationship and those not in a romantic relationship with regards to mask adherence, *Χ*^2^(1) = 1.18, *p* = 0.28.

#### 3.1.3. Limiting Close Contacts

No differences were observed between male and female gender identity in relation to limiting close contacts, *Χ*^2^(1) = 1.35, *p* = 0.25. When comparing those with 10 or fewer close contacts to those with 11 or more close contacts, younger individuals had more close contacts (*M* = 36.04, *SD* = 10.2) than older persons *(M* = 40.92, *SD* = 12.07), *t*(256.47) = −3.56, *p * ≤ 0.001. No differences were observed in years of education between those who reported having less than 10 contacts in the last week at time of assessment and those who reported having more than 10 close contacts in the last week at time of assessment, *t*(260) = −0.84, *p* = 0.40. No differences were observed between White and non-White individuals in limiting close contacts, *Χ*^2^(1) = 0.94, *p* = 0.33. Individuals that identified as Hispanic/Latinx (80.8%) had a greater proportion of 11 or more close contacts in the past week at time of assessment, *Χ*^2^(1) = 11.19, *p* < 0.001, than those that identified as non-Hispanic/Latinx (46.2%). Note that this comparison should be interpreted with caution due to a small number of people identifying as Hispanic/Latinx, which was further divided by ≤10 close contacts (*n* = 21) and ≥11 close contacts (*n* = 5).

No differences were observed in limiting close contacts between those who reported having a combined household income below USD 70,000 and those who reported having a combined household income above USD 70,000, *Χ*^2^(1) = 2.27, *p* = 0.13.

With relation to limiting close contacts, individuals in a romantic relationship (42.4%) had a lower proportion of limiting close contacts at the time of assessment, *Χ*^2^ (1) = 11.77, *p* < 0.001, than those who were not in a romantic relationship (64.5%).

### 3.2. Logistic Regression Analyses

Two logistic regression models examined psychological distress and psychological flexibility processes (openness to experiences, behavioral awareness, valued action) as predictors of adherence to mask-wearing and limiting close contacts, while controlling for demographic correlates.

#### 3.2.1. Mask-Wearing Adherence

Results indicated that greater behavioral awareness significantly predicted greater odds of mask adherence, *Wald*(1) = 10.91, *p* < 0.001, Exp(B) = 1.08, as did lower openness to experiences, *Wald*(1) = 4.72, *p* = 0.03, Exp(B) = 0.96. Female gender identity was a significant covariate, whereas age, perceived stress, and valued action were not significant predictors (see Table 2). Without the components of psychological flexibility, the model accounted for 5% of the variance in mask adherence. With the components of psychological flexibility and distress added in Step 2, the final model accounted for 13% of the variance in mask adherence.

#### 3.2.2. Limiting Close Contacts

Results indicated that greater behavioral awareness predicted greater odds of limiting close contacts, *Wald*(1) = 8.78, *p* = 0.003, Exp(B) = 1.08. Older age and current single romantic relationship status were significant covariates, whereas perceived stress, openness to experiences, and valued action were not significant predictors (see Table 3). Step 1 accounted for 10% of the variance in limiting close contacts. With the components of psychological flexibility and distress added, the final model accounted for 19% of the variance in limiting close contacts.

## 4. Discussion

The goal of the present study was to investigate how psychological flexibility and psychological distress predicted adherence with public health guidelines (mask-wearing, limiting close contacts) during the COVID-19 pandemic. Our results indicated that greater behavioral awareness, a component of psychological flexibility, predicted both greater odds of limiting close contacts and mask-wearing adherence (Table 2 and Table 3). Furthermore, lower openness to experiences related to lower likelihood of mask-wearing, but not limiting close contacts. Valued action was not predictive of adherence with either public health behavior. The results also indicated that nearly one-fifth of the variance in limiting close contacts and 13% of the variance in mask-wearing adherence was accounted for by the predictor variables in the final models.

In an effort to quell community spread of COVID-19, guidelines based on scientific evidence helped to flatten the curve of positive cases and deaths due to COVID-19. These guidelines included wearing a face (mouth and nose) covering when in public, physical distancing (keeping a distance of at least six feet from others while in public), and avoiding public gatherings (i.e., not going to non-essential events/areas that could contribute to the spread of COVID-19). However, adherence to these guidelines varied [6], and the available literature to account for the variability is limited. In Poland, researchers examined the role of personality traits and individual differences as related to perceptions of the COVID-19 pandemic and compliance with the guidelines set in Poland [16], finding that perception of the seriousness of the COVID-19 pandemic played a larger role in variability in compliance than personality traits. This may relate to the result indicating that lower openness to experiences related to lower likelihood of mask-wearing, such that one behavioral indication of low openness to experiences is rule-following behavior or compliance with socially desirable behaviors [9]. The aversive emotions that one may experience when not complying with socially normative and, at times, legally mandated behavior may present a substantial deterrent to non-adherence. This same behavior (mask-wearing) could also serve a values-based function, however, if one connected this behavior to a personal value (e.g., health, community).

Two studies have identified callous traits aligned with psychopathy as being associated with lower compliance [16,17]. Lower levels of empathy were also identified in a Brazilian study with compliance as an outcome [17]. In a sample of individuals from the United States, the United Kingdom, and Germany, empathy related to increased motivation to wear face masks and adhere to physical distancing, and compliance increased when empathy was induced experimentally for those most vulnerable to the most serious consequences of COVID-19 [18]. Empathy can change in accordance with developmental changes and is therapeutically modifiable [19]. Both empathy and perspective-taking are important factors that impact one’s behavioral awareness [20,21]. The identification of both therapeutically modifiable and transdiagnostic processes is critical in the development of targeted interventions that increase the likelihood of adherence to public health behaviors amidst COVID-19. As such, there is opportunity for these constructs to work together under the umbrella of psychological flexibility. For example, a person might become aware of the impact of their behavior on personal and community health, and if either is held as a personal value, they may alter their behavior accordingly, even in the presence of difficult emotions, thoughts, or sensations. Furthermore, while remaining in the present moment and attending to changes in context, they may choose to change behavior as the circumstances change, such as wearing a mask in a hospital but not when outdoors. The findings herein emphasize that behavioral awareness is a particularly important component of psychological flexibility across the adherence variables examined in the context of the COVID-19 pandemic.

Other work has identified psychological flexibility as a key process that impacts mental health and well-being during the COVID-19 pandemic. In a study that focused on the mediating role of psychological flexibility between conspiracy beliefs and lockdown compliance, researchers found that psychological flexibility contributed to greater lockdown compliance when distress regarding the pandemic was reported as low or moderate [22]. Research conducted in the United Kingdom found that psychological flexibility was associated with greater wellbeing, as well as lower anxiety, depression, and distress during the COVID-19 pandemic [23]. In Hong Kong, researchers found that fostering psychological flexibility ultimately mitigated the adverse mental health effects of the COVID-19 pandemic [24]. In Sweden, researchers found that psychological flexibility emerged as a potential protective factor, as it was negatively associated with depression, anxiety, and insomnia [25]. Lastly, in the United States researchers found that individuals with greater psychological flexibility experienced reduced psychological distress during the COVID-19 pandemic [10]. Other studies have further investigated psychological flexibility as it relates to the pandemic in different outcomes, as well as the potentially protective effects of psychological flexibility on mental health during the pandemic [26,27,28,29]. As such, it is clear that psychological flexibility is an important modifiable transdiagnostic predictor of well-being and behavioral compliance during global adversity.

### 4.1. Future Directions

The present study identified psychological flexibility as a process that predicts adherence to public health guidance. In particular, behavioral awareness is a specific component of psychological flexibility that is predictive of increased odds of complying with public health guidance. In reflecting on the adherence to public health behaviors during the COVID-19 pandemic and in looking ahead to limit the impact of future pandemics, it is important to investigate targeted interventions and prevention efforts. Acceptance and Commitment Therapy (ACT) is a transdiagnostic psychotherapy that targets psychological flexibility as the key outcome through interventions focused on acceptance, mindfulness, and behavioral change [9]. As a transdiagnostic intervention, ACT applies across multiple syndromes and is designed for the human condition, not one disorder [9]. ACT targets behavioral awareness, which encourages participation in the present moment, regardless of potential mind-wandering [9]. Behavioral awareness and psychological flexibility more broadly are modifiable processes, and psychological flexibility mediates treatment-related outcomes [9]. In this way, individuals are able to learn how to more effectively respond to difficult situations, making them not only more equipped to handle future challenges, but to lessen the impact of these situations or events [9].

Past work has investigated ACT as a treatment to promote adherence to behaviors in health psychology settings. In a study focusing on the effectiveness of ACT in promoting physical activity, researchers found that study participants in the ACT group significantly increased their physical activity level in comparison to the education-only group [30]. Furthermore, after a telephone-based weight-loss program, the group that received ACT lost more weight when compared to controls [31]. One study focusing on the effect of ACT on diabetes self-management found that, compared to education alone, the group randomized to the ACT condition had overall improved outcomes related to diabetes self-management [32]. Overall, in a review of randomized controlled trials that focused on the effect of ACT on long-term lifestyle and behavioral changes, it was found that the results of the studies suggested that ACT helped to maintain long-term lifestyle and behavioral changes that included weight management, substance and addiction-related issues, and diet and physical activity [33].

Public health guidance to wear a face covering and limit close contacts was received with variable adherence in the US during the pandemic. News stories in the United States detailed protests of masks, and the issue became highly polarized. As such, the messaging around public health guidance may be particularly impactful, but individual differences in response styles may be a target for future interventions. ACT has promoted adherence in several other populations, and thus, may be an appropriate intervention as related to future public health behaviors. The present study indicates that behavioral awareness may be of particular importance in the context of COVID-19.

### 4.2. Limitations

The findings should be considered along with several limitations. This study relied on self-report measures, which introduce social desirability biases that are relevant to adherence behaviors. However, adherence to public health guidelines could not be measured behaviorally or by collateral report during a time when limiting close contacts was encouraged or mandated. To increase likelihood of valid responses, surveys were entirely anonymous. Furthermore, the majority of the participants reported having a full-time job at the time of survey completion, which could lead participants to have other, external factors in mind, such as maintaining their job when responding to questions regarding mask usage and number of close contacts within the past week. Study participants who completed all of the assessments (as compared to those who did not complete all assessments) were more highly educated and more likely to be non-Hispanic, and our results may only generalize to a sample similar to this one. Additionally, study participants were recruited via MTurk. Data integrity concerns regarding MTurk have been previously stated due to the fact that non-human responders have been found to participate intermittently [34]. However, attention checks and Google reCAPTCHA were utilized in the present study in an effort to ensure valid, human responses. Furthermore, MTurk was utilized due to the ability to reach a more diverse sample, as researchers have found that MTurk samples are both more demographically diverse in comparison to typical internet samples, as well as American college samples [35,36]. Additionally, there were external variables that were not controlled for, such as cultural beliefs that might vary by culture and community. Future research should examine cross-cultural adherence to public health guidelines, particularly in the context of a global pandemic. Furthermore, one of the key questions in this study focused on mask-wearing in public spaces, but as the study questions were answered via self-report, what an individual deemed as “public” could have been subjective and interpreted differently by participants. Additionally, income and relationship status were measured at the July 2020 timepoint, so these variables could not be included in the attrition analyses. Finally, there was participant attrition between time points, as 95 participants were lost been the June and July 2020 assessments; however, the study remained sufficiently powered for analyses.

## 5. Conclusions

The COVID-19 pandemic brought many global changes and took hundreds of thousands of lives. Two prominent and widespread behavioral changes in the United States included mask-wearing and limiting close contacts, which were directives issued by public health authorities early in the pandemic to slow community spread. Despite strong scientific evidence for each, adherence was variable, and the cost in human lives and health was high. The present study identified behavioral awareness as a key process that predicted greater odds of mask-wearing adherence and limiting close contacts. As a component of psychological flexibility, behavioral awareness represents the ability to stay in the moment and intentionally choose how to respond to changing contexts. This is a modifiable, transdiagnostic skill that can be improved through use of ACT and other mindfulness interventions. As such, behavioral awareness represents a possible target for future interventions to improve adherence behaviors, particularly those that impact not only one’s own health, but the health of the global community.

## Figures and Tables

**Table 1:** Descriptive characteristics of the sample, *n* = 265.

	*n* (%)
Age, M(SD)	38.49 (11.42)
Years of education, M(SD)	15.35 (2.53)
Race	
White	208 (78.8%)
African American or Black	32 (12.1%)
American Indian or Alaska Native	5 (1.9%)
Asian	12 (4.5%)
Biracial or Multiracial	7 (2.7%)
Did not disclose	1 (0.4%)
Ethnicity	
Non-Hispanic	238 (90.2%)
Hispanic	26 (9.8%)
Did not disclose	1
Gender identity	
Female	104 (39.4%)
Male	158 (59.8%)
Transgender man	1 (0.4%)
Self-described	1 (0.4%)
Prefer not to disclose	1 (0.4%)
Other Covariates	
In a romantic relationship	172 (64.9%)
Household income of ≤$70,000	184 (69.4%)

M = Mean, and this is italicized per APA style. SD = Standard Deviation.

**Table 2:** Mask adherence, as predicted by psychological flexibility components and perceived stress.

	B	SE	Wald	*p*	Exp(B)	CI for Exp(B)	Nagelkerke’s Pseudo-R^2^
Step 1							0.05
**Gender identity**	**−0.76**	**0.28**	**7.21**	**0.007**	**0.47**	**0.27–0.82**	
Age	0.01	0.01	0.28	0.60	1.01	0.98–1.03	
Step 2							0.13
**Openness to experiences**	**−0.04**	**0.02**	**4.72**	**0.03**	**0.96**	**0.93–1.0**	
**Behavioral awareness**	**0.08**	**0.02**	**10.91**	**<0.001**	**1.08**	**1.03–1.14**	
Valued action	−0.01	0.02	0.29	0.59	0.99	0.96–1.03	
Perceived stress	−0.03	0.02	1.53	0.22	0.97	0.93–1.02	

Note: Gender identity was coded as 0 = female-identifying, 1 = male-identifying. Openness to experiences, behavioral awareness, and valued action were measured using the Comprehensive Assessment of Acceptance and Commitment Therapy processes. Perceived stress was measured using the Perceived Stress Scale-10. Bolded rows signify statistically significant findings.

**Table 3:** Limiting close contacts adherence, as predicted by psychological flexibility components and perceived stress.

	B	SE	Wald	*p*	Exp(B)	CI for Exp(B)	Nagelkerke’s Pseudo-R^2^
Step 1							0.10
**Relationship status**	**−1.10**	**0.30**	**13.49**	**<0.001**	**0.33**	**0.19–0.60**	
**Age**	**0.04**	**0.01**	**7.04**	**0.008**	**1.04**	**1.01–1.06**	
Step 2							0.19
Openness to experiences	−0.01	0.02	0.17	0.68	0.99	0.96–1.03	
**Behavioral awareness**	**0.08**	**0.03**	**8.78**	**0.003**	**1.08**	**1.03–1.13**	
Valued action	0.002	0.02	0.01	0.93	1.00	0.97–1.04	
Perceived stress	−0.01	0.02	0.13	0.72	0.99	0.95–1.04	

Note. Relationship status was coded as 0 = single, 1 = in a relationship. Openness to experiences, behavioral awareness, and valued action were measured using the Comprehensive Assessment of Acceptance and Commitment Therapy processes. Perceived stress was measured using the Perceived Stress Scale-10. Bolded rows are statistically significant.

## Data Availability

The IRB approved data sharing in de-identified and aggregated form after request by interested researchers.
